# Population origin determines the adaptive potential for the advancement of flowering onset in *Lupinus angustifolius* L. (Fabaceae)

**DOI:** 10.1111/eva.13510

**Published:** 2022-11-29

**Authors:** Sandra Sacristán‐Bajo, Alfredo García‐Fernández, Carlos Lara‐Romero, Samuel Prieto‐Benítez, Pablo Tabarés, Javier Morente‐López, María Luisa Rubio Teso, Aitor Alameda‐Martín, Elena Torres, José María Iriondo

**Affiliations:** ^1^ Grupo de Ecología Evolutiva (ECOEVO), Área de Biodiversidad y Conservación, Dpto. de Biología, Geología, Física y Química Inorgánica, ESCET Universidad Rey Juan Carlos Madrid Spain; ^2^ Área de Botánica, Facultad de Farmacia Universidad Complutense Madrid Spain; ^3^ Unidad de Ecotoxicología de la Contaminación Atmosférica, Departamento de Medio Ambiente CIEMAT Madrid Spain; ^4^ Grupo de Investigación de Ecología y Evolución en Islas Instituto de Productos Naturales y Agrobiología (IPNA‐CSIC) Tenerife Spain; ^5^ Departamento de Agronomía Universidad de Almería Almería Spain; ^6^ Departamento de Biotecnología‐Biología Vegetal Universidad Politécnica de Madrid Madrid Spain

**Keywords:** artificial selection, climate change, evolutionary ecology, manual crosses, plant traits, population origin

## Abstract

In the present framework of global warming, it is unclear whether evolutionary adaptation can happen quick enough to preserve the persistence of many species. Specifically, we lack knowledge about the adaptive potential of the different populations in relation to the various constraints that may hamper particular adaptations. There is evidence indicating that early flowering often provides an adaptive advantage to plants in temperate zones in response to global warming. Thus, the objective of this study was to assess the adaptive potential for advancing flowering onset in *Lupinus angustifolius* L. (Fabaceae). Seeds from four populations from two contrasting latitudes in Spain were collected and sown in a common garden environment. Selecting the 25% of the individuals that flowered earlier in the first generation, over three generations, three different early flowering selection lines were established, involving both self‐crosses and outcrosses. All artificial selection lines advanced their flowering significantly with respect to the control line in the northernmost populations, but not in the southern ones. Selection lines obtained from outcrossing had a greater advancement in flowering than those from self‐crossing. No differences were found in the number or weight of the seeds produced between control and artificial selection lines, probably because plants in the common garden were drip irrigated. These results suggest that northern populations may have a greater adaptive potential and that southern populations may be more vulnerable in the context of climate warming. However, earlier flowering was also associated with changes in other traits (height, biomass, shoot growth, specific leaflet area, and leaflet dry matter content), and the effects of these changes varied greatly depending on the latitude of the population and selection line. Assessments of the ability of populations to cope with climate change through this and other approaches are essential to manage species and populations in a more efficient way.

## 1 INTRODUCTION

Species possess different strategies to cope with climate change. One of the best documented responses is that species distributions are shifting towards higher latitudes and/or elevations to occupy areas within their ranges of thermal tolerance (Forero‐Medina et al., 2011; Parmesan & Yohe, 2003; Root et al., 2003). However, some species will not be able to migrate fast enough to avoid extinction, especially those that have limited dispersal capacity, such as many plants (Berg et al., 2010). In these cases, phenotypic plasticity and adaptive evolutionary responses are of vital importance (Jump & Peñuelas, 2005; Teplitsky & Millien, 2014). The ability of species to evolve in response to environmental changes constitutes their adaptive or evolutionary potential (Funk et al., 2019). Given the exceptional swiftness of climate change (Shaw & Etterson, 2012), it is unclear for many species whether evolutionary adaptation can occur fast enough to ensure population survival. Therefore, characterizing adaptive potential is essential for assessing the resilience and extinction risk of species and populations to climate change. Nevertheless, it can be a very difficult feature to measure and quantify (Funk et al., 2019; Williams et al., 2008).

Artificial selection is a useful way to test a species' evolutionary potential and determine the nature and strength of its evolutionary constraints (Conner, 2003; Hoffmann & Sgró, 2011). It is a process in which humans select individuals of a given species with certain phenotypic traits for breeding, to enhance and perpetuate those traits in future generations (Conner, 2016). It has been used to improve many different traits during the domestication of crops, livestock, and pets, such as changes in size, shape, or color, adaptation to environmental conditions, or resistance to pests and diseases (Conner, 2016; Dempewolf et al., 2014). However, the use of artificial selection in the fields of conservation biology and adaptive evolution has been so far little explored (Selwood et al., 2015).

Artificial selection can provide estimates of the magnitude of additive genetic variance for a trait and the genetic covariance between the selected trait and other traits. The additive genetic variance of a trait is essential for natural selection to act upon and bring about evolutionary change. On the contrary, genetic covariances with other traits are also important because, through them, natural selection on one trait causes an evolutionary change in a correlated trait, which may or may not be itself under direct selection (Conner, 2003). In the former case, genetic covariances can explain the existence of trade‐offs between fitness‐related traits (Etterson & Shaw, 2001; Walsh & Blows, 2009; Worley & Barrett, 2000). Therefore, artificial selection can be a sound way to determine how a single trait may evolve under a given strength of natural selection.

Since environmental pressures may vary among populations, a given trait can acquire different values through local adaptation depending on the population origin (Debieu et al., 2013; Milla et al., 2009; Morente‐López et al., 2020). Thus, the differences in genetic diversity within and between populations influence the adaptive potential of a species (Funk et al., 2019). Conducting artificial selection experiments on populations originating from different environmental conditions can inform us about the strength and speed with which a trait can evolve in response to environmental changes in each population and which populations have greater adaptive potential for a given trait (Conner, 2003). In this context, they can be used to determine the vulnerability of populations to climate change and to explore how we can act to mitigate their effects.

One of the consequences of global warming is the early arrival of spring and the late arrival of winter in temperate zones, which has prompted the modification of phenological traits in many species (Bradshaw & Holzapfel, 2008). For instance, in plant species, the transition from the vegetative to the reproductive phase is a crucial step in their life cycle (Blümel et al., 2015) and the timing of reproduction greatly influences reproductive success (Forrest & Thomson, 2010; Landa, 1992; Thomas et al., 2001). There is already considerable evidence that climate change is favoring advanced flowering plants in temperate zones (Büntgen et al., 2022; Fitter & Fitter, 2002; Peñuelas et al., 2002) and that flowering time is a highly heritable character (Franks et al., 2007). Therefore, flowering time is a key trait for plant adaptation to climate change (Franks & Hoffmann, 2012). The proper synchronization of flowering time with ideal environmental conditions is a delicate task that implies the integration of numerous external and internal signals (Putterill et al., 2004). Thus, the control of flowering time entails a complex network of different genetic and epigenetic regulators (Blümel et al., 2015), implying that it is a polygenic trait with more than 100 implicated genes identified in some species (Blümel et al., 2015; Weller & Ortega, 2015).

The main objective of our study was to use artificial selection to assess the adaptive potential for advancing flowering onset in two sets of populations of contrasting origins of the annual legume *Lupinus angustifolius* L. We hypothesized that, given the polygenic nature of the trait, there would be substantial standing genetic variation to obtain artificially selected subsets whose progeny would flower significantly earlier than the population mean, even in a selfing species such as *L. angustifolius*. Moreover, forcing the outcrossing between the artificially selected individuals might generate greater genetic variation and phenotypes that flower even earlier. Considering that the two sets of populations differ in the temperature regimes of their localities of origin (southern populations have warmer temperature regimes), we expected that flowering time may advance less in southern populations with warmer conditions, because natural selection may have already acted in these populations to select for early flowering genotypes, thus reducing available genetic variation. Additionally, there are potential epistatic and pleiotropic interactions between the genes involved in the regulation of flowering time and those involved in the expression of other traits. Thus, we hypothesized that artificial selection to advance the onset of flowering might indirectly affect the expression of other plant traits, which may also contribute to the overall fitness of the individuals. Considering the “fast‐slow” plant economics spectrum that integrates traits through leaves, stems, and roots and results in the existence of trade‐offs between traits (Reich, 2014), we would expect that plants that flower earlier would have lower biomass and growth, since they would allocate more resources to reproduction. Using a common garden experiment, we compared early flowering selection lines, obtained through self‐crosses or outcrosses, against a control line in four populations of two climatically contrasted regions in the Iberian Peninsula. We recorded flowering onset and measured a suite of other plant traits to answer the following questions: (i) Is there adaptive potential for advanced flowering in the study populations of *L. angustifolius*? (ii) Can outcrossing of the artificially selected subset advance further flowering onset? (iii) Is the intensity of flowering onset advance dependent on the population of origin or the latitude of the material subjected to artificial selection? (iv) Which other traits will be affected by advancing flowering onset and how?

## 2 MATERIALS AND METHODS

### 2.1 Study species and collected material

*Lupinus angustifolius* L. (Fabaceae) is an annual herbaceous plant that occurs in the Mediterranean Region and has been introduced as a cultivated crop all around the world (Castroviejo & Pascual, 1993). The flower is hermaphroditic and mostly self‐pollinates before its petals open (Wolko et al., 2011). Natural outcrossing estimates are below 2% (Dracup & Thomson, 2000). The inflorescence can have up to 30 violet flowers developing acropetally and produces pods with 3–7 seeds (Clements et al., 2005). Flowering onset is controlled by photoperiod and vernalization, and long days accelerate the start of flowering (Gladstones & Hill, 1969; Rahman & Gladstones, 1974).

**FIGURE 1:**
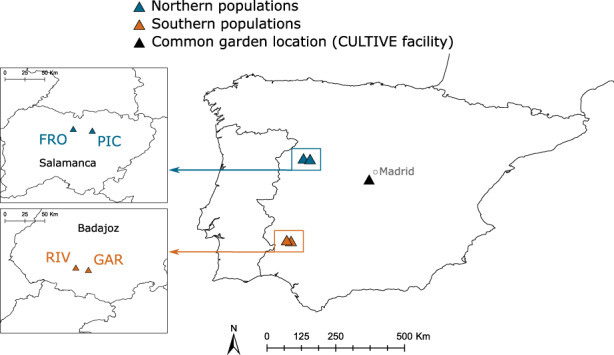
Location of northern (blue) and southern (orange) populations of *Lupinus angustifolius* L. in the Iberian Peninsula. A detail of the populations and the provinces in which they are situated is shown in the squares on the left.

In the summer of 2016, we collected seeds from four populations of *L. angustifolius* located in Central and Southern Spain (Table 1, Figure 1). All the populations had a large number of individuals (more than 500). The two populations within each region are located less than 20 km apart, whereas the distance between northern and southern populations is approximately 300 km. In each population, we separately collected seeds from 98 mother plants (genotypes) that were located at least 1 m apart from each other.

**TABLE 1:** Populations of *Lupinus angustifolius* L. and common garden site involved in the study.

Acronym	Town	Region	Latitude	Longitude	Elevation (m. a.s.l.)	Annual mean temperature (°C) and coefficient of variation (in brackets)	May–July precipitation (mm) and coefficient of variation (in brackets)
FRO (N)	Zafrón	Central Spain	41.0241	−6.0281	840	12.4 (3.2)	92 (46)
PIC (N)	Zarapicos	Central Spain	41.0043	−5.8130	820	12.6 (3.1)	89 (45)
GAR (S)	La Garranchosa	Southern Spain	38.3257	−6.4337	422	16.5 (2.4)	64 (64)
RIV (S)	Rivera de la Lanchita	Southern Spain	38.3515	−6.5760	352	16.8 (2.2)	61 (63)
—	Common garden 2017	Central Spain	40.3343	−3.8829	690	15.1	72
—	Common garden 2018	Central Spain	40.3343	−3.8829	690	14.6	95
—	Common garden 2019	Central Spain	40.3343	−3.8829	690	14.8	16
—	Common garden 2020	Central Spain	40.3343	−3.8829	690	15.1	69

*Note*: Town, region, geographical coordinates (decimal degrees, WGS84), and climate variables associated with the populations (1985–2015 period) and to the common garden site (years 2017–2020). May–July period corresponds with the period when the plants are developing fruits and setting seeds. Climate data were obtained from ClimateEU (Marchi et al., 2020).

Abbreviations: FRO, Zafrón; GAR, La Garranchosa; PIC, Zarapicos; RIV, Rivera de la Lanchita.

### 2.2 Cultivation process and artificial crosses

In the autumn of 2016, seeds from the 98 collected genotypes of each population were sown in a greenhouse at the CULTIVE facility (https://urjc‐cultive.webnode.es/) at Rey Juan Carlos University (Móstoles, Madrid). The facility is located in Central Spain, at an intermediate elevation, latitude, and temperature regime between the two sets of populations studied (Table 1, Figure 1). The temperature range inside the greenhouse varied between 1 and 25°C, and plants were only exposed to natural light. Seeds were previously scarified by clipping a small fraction of the seed coat to ensure germination and deposited in 6 L pots with a mixture of 50% sand and 50% commercial substrate enriched with NPK (Klasmann). For each maternal genotype of each population, two pots were assigned, and three seeds were sown in each, constituting a total of 784 pots. When seeds germinated, one seedling was left in each pot and the rest were clipped. In February 2017, the pots were transferred out of the greenhouse and moved outdoors. Pots were randomly distributed following a block design where populations were evenly represented in each block and regularly watered with drip irrigation to constitute a common garden environment.

In the spring of 2017, the flowering phenology of plants was monitored daily. Flowering onset was calculated as the number of days from the sowing date to the appearance of the first flower of the plant. We considered that each plant had flowered when blue‐purple petals of one flower in the main inflorescence could be clearly seen. Artificial selection was implemented one single time as follows: for each population, the plant genotypes that flowered earlier (approximately the first quartile of flowering onset) were tagged and their seeds were separately collected to create an early flowering selection line (hereafter, EFL). For each population, we also generated a control line (hereafter, control flowering line [CFL]) by randomly selecting another 25% among all 98 genotypes. Once again, seeds of each genotype were separately collected and stored. In the autumn of 2017, the seeds from the chosen genotypes of early flowering selection lines and from the control lines of each population were scarified and sown in pots in the greenhouse as described above. This process was repeated in the same way in subsequent culture cycles in the 2017–2018, 2018–2019, and 2019–2020 seasons. However, in the early flowering selection line, no further selection was implemented. Instead, in the EFL and CFL, seeds of each genotype cultivated in the previous season were separately collected and stored, until use in the subsequent season. In the seasons 2017–2018 and 2018–2019, each genotype was replicated in four pots. In 2019–2020, each genotype was replicated in just two pots.

In the spring of 2018, we manually crossed genotypes from the early flowering selection line (EFL) between each other to obtain an outcrossed early flowering line (hereafter, outbred line [OUT]). Flowering individuals from the EFL were randomly paired, and manual crosses were carried out in both directions following the protocol described in Appendix S1.

**FIGURE 2:**
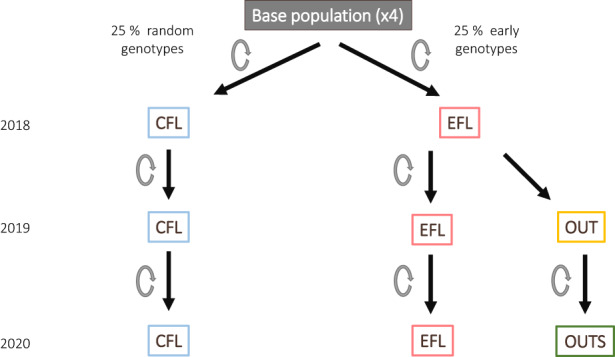
Flowchart depicting the process for generating the different lines. Gray circles next to the arrows indicate that the individuals of that line were self‐crossed. CFL, control flowering line; EFL, early flowering line (self‐pollinated); OUT, outbred line (cross of different EFL genotypes); OUTS, F2 generation of outbred line resulting from the self‐pollination of OUT genotypes.

In the flowering season of spring 2019, we let the individuals of the OUT line self‐pollinate, generating a segregating F2 line (hereafter, self‐crossed outbred line [OUTS]). The OUT line is potentially highly heterozygous and cannot be maintained in a naturally autogamous species. Therefore, it is important to see what happens to the focal trait in subsequent generations when the natural self‐pollination process is resumed. Figure 2 shows a diagram of the complete process. The information on the sample sizes per population for each selection line and year at the flowering time is provided in Table S1.

### 2.3 Trait measurement

Flowering onset was monitored daily for all plants. At the end of each season, the scars that flower peduncles left in the stalks were counted to estimate the number of fruits per plant. The number of seeds per plant was estimated by multiplying the number of fruits of each plant by the average number of seeds per fruit. The average number of seeds per fruit was calculated from the count of the seeds present in up to 15 randomly chosen fruits in each plant. Mean seed weight was estimated from the individual weight of 10 randomly chosen seeds of each plant. Number of seeds per plant and mean seed weight were considered proxies for measuring fitness. Germination rates were not included because they are close to 100% when seeds are scarified. Similarly, the survival rate of plants under controlled conditions is also close to 100%.

To measure the specific leaflet area (SLA) and leaflet dry matter content (LDMC), the central leaflets from eight different fully developed leaves belonging to the lateral branches were collected. The fresh leaflets were weighed immediately in a Kern ABJ 120‐4M analytical balance (Kern & Sohn GmbH). After that, leaflets were placed in water‐saturated filter paper and stored in plastic bags and then refrigerated overnight at 4°C. The next day, we weighed the leaflets again to obtain the saturated weight and measured the foliar area of the leaflets using a foliar scanner Li‐3000C (Li‐Cor). Finally, leaflets were dried in an oven at 60°C for at least 72 h, and then they were weighed to obtain the dry weight. SLA was calculated by dividing the foliar area of a leaflet by its dry weight (Rosbakh et al., 2015). LDMC was calculated by dividing the leaflet dry weight by their saturated weight (Wilson et al., 1999). Total plant height was considered as the distance from the base of the plant to the edge of the main inflorescence at the end of the flowering season. Additionally, we measured the length of the plant from its base to the first flower at the beginning of flowering onset and at the end of the season, on the same dates for all the plants of the experiment. Consequently, we estimated shoot growth as the difference between these two measurements. We also measured the aboveground biomass of each plant at the end of the season using the balance previously mentioned.

In 2018 and 2019, we measured all the traits described above except shoot growth, which was only measured for the year 2019. In the year 2020, due to the pandemic lockdown, we only measured the flowering onset. For this reason, we have characterized the four lines for flowering onset for each population, but only three lines for the remaining traits.

### 2.4 Data analyses

Following Conner (2003), the selection differential was calculated by subtracting the 2017 flowering initiation means of CFL and EFL. Similarly, the response to selection was calculated as the difference between the population means of CFL and EFL in the next generation (2018). The ratio between the response to selection and the selection differential indicates the heritability of the trait in each population. These parameters were calculated to assess how much genetic variation the trait studied has in each population, and how strongly it responded to the selection exerted on them (Conner, 2003).

To assess the differences in flowering onset between populations occurring at the two latitudes prior to selection, we calculated the F statistic applying linear models by using the *lm* function implemented in R version 4.1.1. (http://r‐project.org). To test the effect of artificial selection on flowering onset, we calculated the chi‐squared statistic of the Type II Wald chi‐square tests using generalized linear mixed models (hereafter, GLMMs), and to assess their effect on fitness and morphological traits, we calculated the chi‐squared statistic using linear mixed models (hereafter, LMMs). Generalized linear mixed models (GLMMs) and linear mixed models (LMMs) were fitted using the *glmer* and *lmer* functions included in the R package lme4 version 1.1‐27.1 (Bates et al., 2015). Models were run separately for each response variable. Due to the substantial similarities in response between the populations from the same latitude, we created a new variable called “latitude” to group the two northern and the two southern populations. We included *selection line* (CFL, EFL, OUT, and OUTS), *year* (2018, 2019, and 2020), and *latitude* (North *vs*. South) as fixed effects, and *genotype* and *population* as random effects. Initially, for all the response variables, we tested the interaction between the variables *line* and *year*. Except for SLA and LDMC, this interaction was not significant, so it was excluded from the models. For SLA and LDMC, analyses were performed separately for each year. In all models, we have also included the interaction between *latitude* and *line*. We used a Poisson distribution for the flowering onset variable and a Gaussian distribution for the rest of the traits. Model residuals were checked graphically for normality and homogeneity of variances using diagnostic plots. *R* ^2^ values were calculated using the *summ* function from the package jtools version 2.2.0 (Long, 2019). The significance of each fixed effect was quantified using the *Anova* function from R package car version 3.0‐11 (Fox & Weisberg, 2011). Differences between lines were calculated using Tukey post hoc analyses from R package emmeans version 1.6.3 (Lenth, 2019). Posterior mean values, standard errors, and 95% credible intervals for the different traits and lines were also calculated with the emmeans package. Correlations for the control line in the year 2019 between flowering onset and the rest of the traits were performed using the *corrplot* function from R package corrplot version 0.90 (Wei et al., 2017).

## 3 RESULTS

### 3.1 Flowering onset

**FIGURE 3:**
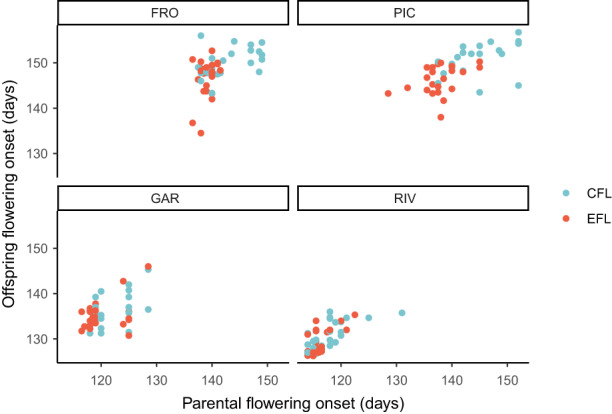
Selection differential for the flowering onset of the season 2017–2018 for the four populations of *Lupinus angustifolius* L. CFL, control flowering line; EFL, early flowering line (self‐pollinated).

In 2017, the first generation of plants grown in the common garden showed that the onset of flowering of plants from the northern populations (140 ± 12 days) occurred significantly later than that of southern populations (121 ± 10 days; *F* = 1006.7, *p* < 0.001, df = 1) (Figure S1). The statistical analysis covering the common gardens in the years 2018, 2019, and 2020 also showed a similar pattern (*X* ^2^ = 41.841, *p* < 0.001, df = 1). The artificial selection applied to obtain the EFL revealed that the selection differential, the response to selection, and the heritability of the flowering onset were higher in the northern populations (Table 2, Figure 3).

**TABLE 2:** Selection differential, response to selection, and heritability for the flowering onset of the season 2017–2018 for the four studied populations of *Lupinus angustifolius* L.

Population	Selection differential	Response to selection	Heritability
FRO	4.481443	1.7420213	0.3887188
PIC	5.371562	2.0188889	0.3758477
GAR	2.905329	0.6503221	0.2238377
RIV	2.924414	0.8248611	0.2820603

**FIGURE 4:**
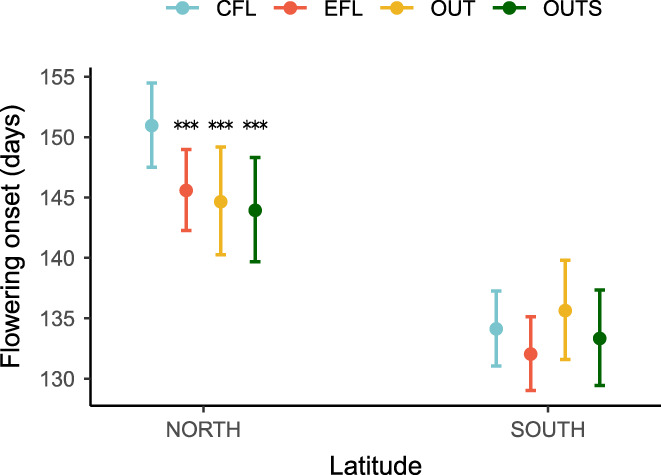
Effect of artificial selection lines (EFL, OUT, and OUTS) on advancing flowering onset of *Lupinus angustifolius* L. Populations are grouped by latitude. Years are analyzed together. CFL, control flowering line; EFL, early flowering line (self‐pollinated); OUT, outbred line (cross of different EFL genotypes); OUTS, F2 generation of outbred lines resulting from the self‐pollination of OUT genotypes. Dots and bars represent the predicted mean from the GLMM model with a Poisson distribution and the 95% confidence intervals. Significant differences (*p* < 0.05) determined by Tukey test between artificial selection lines and the control line are marked with asterisks (*p < 0.05; **p < 0.01; ***p < 0.001).

The interaction between latitude and line was statistically significant (*X* ^2^ = 17.298, *p* < 0.001, df = 3), indicating that artificial selection significantly modified flowering onset in northern but not in southern populations (Figure 4, Tables S2 and S3). Thus, the EFL, OUT, and OUTS lines from the northern populations advanced their flowering time by an average of 6, 16, and 21 days, respectively, compared with the control line (Table S5). Fixed effects explained 59% of the variation in flowering onset, whereas random effects explained 1.3% (Table S2). Flowering time for each year, population, and line is shown in Figures S2–S4. Posterior mean values, standard errors, and 95% confidence intervals for each line are shown in Table S4.

### 3.2 Reproductive success

Artificial selection did not result in differences in reproductive success among lines, that is, number of seeds (*X* ^2^ = 0.254, *p* = 0.881, df = 2) and seed weight (*X* ^2^ = 3.983, *p* = 0.137, df = 2) (Figure S5, Tables S2 and S3) in any of the latitudes. However, significant differences were found in the number (*X* ^2^ = 22.800, *p* < 0.001, df = 1) and in the weight (*X* ^2^ = 61.890, *p* < 0.001, df = 1) of the seeds between latitudes (Tables S2 and S3). Northern population plants produced a greater number of seeds than southern populations, but the weight of their seeds was lower. Fixed effects explained 19% of the variation in the number of seeds and 50% in seed weight, whereas random effects explained 3.4% and 13%, respectively (Table S2). Posterior mean values, standard errors, and 95% confidence intervals for the number of seeds and seed weight for each line are shown in Table S4.

### 3.3 Correlated responses to selection for early flowering

**FIGURE 5:**
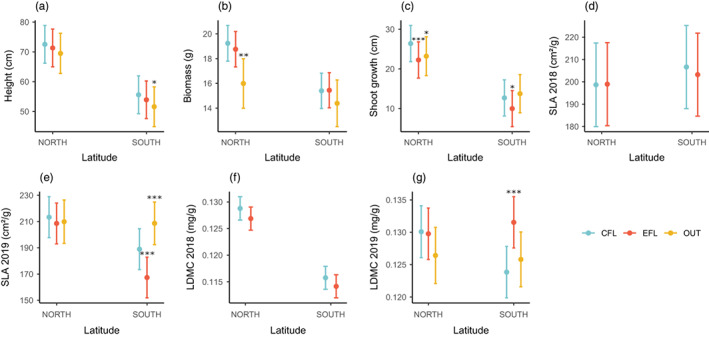
Effect of artificial selection lines (EFL, OUT, and OUTS) on other functional traits: (a) plant height, (b) biomass, (c) shoot growth, (d) specific leaflet area (SLA) 2018, (e) specific leaflet area (SLA) 2019, (f) leaflet dry matter content (LDMC) 2018, and (g) leaflet dry matter content (LDMC) 2019. Years are analyzed together except for SLA and LDMC. CFL, control flowering line; EFL, early flowering line (self‐pollinated); OUT, outbred line (cross of different EFL genotypes). Dots and bars represent the predicted mean from the LMM model with a Gaussian distribution and the 95% confidence intervals. Significant differences (*p* < 0.05) determined by Tukey test between artificial selection lines and the control line are marked with asterisks (*p < 0.05; **p < 0.01; ***p < 0.001).

The effect of EFL was significantly associated with lower shoot growth in both northern and southern populations and higher LDMC and lower SLA (year 2019) in southern populations (Figure 5c,e,g). The effect of the OUT line was significantly associated with shorter height and higher SLA (2019) in southern populations and lower biomass and shoot growth in northern populations (Figure 5). A secondary, but relevant, result from the analyses performed on plant height, biomass, and shoot growth is that plants from the northern populations were significantly taller and had higher biomass and shoot growth than those from the southern populations (All *X* ^2^ test: *p* < 0.001; Figure 5, Tables S2 and S3). For all studied traits, fixed effects explained between 41% and 0.7% of the variance and random effects, between 29.6% and 5.1% (Table S2). Posterior mean values, standard errors, and 95% confidence intervals for the different plant traits for each line are shown in Table S4.

**FIGURE 6:**
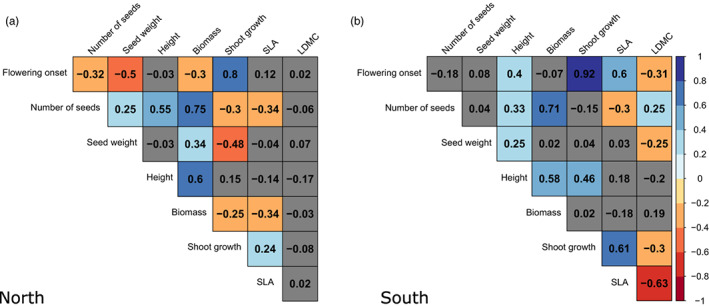
Correlations between flowering onset and other traits for control flowering line (CFL) and year 2019. (a) Correlations for northern latitude populations. (b) Correlations for southern latitude populations. Positive correlations are represented in cold colors, while negative correlations are represented in warm colors. Nonsignificant correlations (*p* > 0.05) are represented in gray.

Several significant correlations were also found between flowering onset and other traits for CFL and year 2019. In northern populations, plants that flowered earlier showed an increase in their biomass, seed number, and seed weight, and a decrease in shoot growth (Figure 6a). In southern populations, plants that flowered earlier showed a reduction in height, shoot growth and SLA, and an increase in LDMC (Figure 6b).

## 4 DISCUSSION

The offspring of artificial selection lines experienced an advance in the flowering onset of *L. angustifolius*. However, the likelihood and magnitude of this change depended largely on the precise environmental conditions experienced by the populations in their native locations and the type of crosses applied (selfing or outcrossing). Thus, flowering onset was significantly modified in the northern populations but not in the southern ones, indicating a greater adaptive potential of the former regarding this trait. The selection lines that were obtained through forced outcrossing between early flowering plants generated plants that flowered even earlier, disclosing the involvement of numerous alleles from multiple genes in the configuration of early flowering phenotypes. Furthermore, advancing flowering onset by artificial selection was associated with changes in other plant traits that varied depending on the latitude of the populations of origin, potentially leading to constraints to adaptation.

### 4.1 Advancement of flowering onset

All artificial selection lines flowered significantly earlier than the control line in the northern populations. However, this did not occur in the southern populations. The OUT and the OUTS of the northern populations advanced their flowering onset by a greater number of days (16 and 21 days, respectively) than the self‐crossed EFL, which advanced 6 days with respect to control. The greater advance of OUT and OUTS lines may derive from new allelic combinations that arise from the outcrossing of EFL individuals, which, due to the autogamous nature of the plant, are likely to be homozygous in most loci. This indicates that not only the trait is heritable, as confirmed by the estimates obtained in our experiment, but also numerous different alleles from multiple genes are involved in the configuration of early flowering phenotypes. The range of flowering onset advance obtained is comparable to those obtained in similar studies where artificial selection was used. For example, Burgess et al. (2007), working with *Campanulastrum americanum* (L.) Small, obtained an early flowering line through manual crosses between early flowering plants. After three generations, flowering onset was advanced by an average of 13 days compared with the control line. Similarly, Sheth and Angert (2016) obtained an early flowering selection line in *Mimulus cardinalis* Benth that flowered 15 days earlier than the control line after two generations. These two species are xenogamous, whereas *L. angustifolius* is an almost strict autogamous species (Wolko et al., 2011), and thus, one would expect *L. angustifolius* populations to have less standing genetic variation. However, this apparent limitation did not prevent a similar advance in flowering time when compared to the abovementioned outcrossing species.

In contrast to our results, Sheth and Angert (2016) reported that populations from higher latitudes of *M. cardinalis* flowered earlier than those from lower latitudes and that the latter presented a response of greater magnitude to artificial selection. The apparently contrasting responses found in *L. angustifolius* and *M. cardinalis* can be explained by the different limiting factors operating in each case. While in the former, hot dry summers force warm populations to flower earlier to complete the reproductive process, in the latter, strong selection on flowering time takes place at the high‐latitude populations to ensure that plants mature fruits before the growing season ends, in this case, due to the arrival of frosts (Munguía‐Rosas et al., 2011; Sheth & Angert, 2016).

The evolutionary potential of some populations to respond to environmental changes may be limited by the lack of genetic variation (Sheth & Angert, 2016). For example, in our experiment, the lack of advancement in flowering time through artificial selection in the southern populations along with their earlier flowering time compared with northern populations suggests that these populations had already experienced prior natural selection for early flowering due to the higher temperatures and lower interannual variation in temperatures occurring at their locations (Table 1). Consequently, their standing genetic variation for early flowering would be currently lower than that found in the northern populations. Flowering onset may be under weaker selection in northern populations because the growing season is longer due to a later arrival of the drought (Table 1, Matesanz et al., 2020). The lower selection differentials and responses to selection obtained in the southern populations also support this idea (Table 2). These results suggest that northern populations of *L. angustifolius* have a greater adaptive potential and could evolve more rapidly toward earlier flowering phenotypes in response to global warming.

### 4.2 Effect on plant reproductive success

Although early flowering phenotypes are expected to be associated with an increase in reproductive success in temperate zones in response to current global warming (Munguía‐Rosas et al., 2011), we did not find an association between selection lines and fitness components (seed number and seed weight) in our study. This is probably explained by the fact that our experiment provided water on demand by drip irrigation throughout the life cycle of the plants and that water availability is the main constraint for plant growth and performance for plants, like *L. angustifolius*, that occur in the Mediterranean region (Blondel et al., 2010; Matesanz et al., 2020; Matesanz & Valladares, 2014). In any case, the effects of early flowering on fitness components may not be straightforward and may involve changes in both seed number and size. For instance, the advance in flowering date might be associated with a reduction in seed number, but increase in seed weight, as we observed in our experiment by the differences in these traits between southern and northern populations (the former flowering earlier than the latter). The trade‐off between number and seed size has been extensively studied (see Lázaro & Larrinaga, 2018). Producing a greater number of seeds can provide a greater number of offspring and greater reproductive success, whereas larger seeds may ensure the survival of individuals. Therefore, individuals from stable or resource‐rich environments may benefit by producing more seeds, while those from more unstable or resource‐poor environments may be more successful by producing fewer but heavier seeds (Leishman et al., 2009; Metz et al., 2010). For instance, Burgess et al. (2007) in their artificial selection greenhouse experiment with *C. americanum* observed that the early flowering lines produced fewer seeds but yielded greater seed weight than the late flowering lines. However, working with the same populations used in our study, Matesanz et al. (2020) observed that southern populations of *L. angustifolius* (which flower earlier) produced more and heavier seeds than northern populations when plants were subjected to a drought treatment. In view of these results, a different experiment involving the sowing of control and artificial selection lines in natural conditions near the original populations would be needed to thoroughly test the fitness benefits of early flowering plants.

### 4.3 Association with other traits

Artificial selection has the potential to modify the trait of interest within a few generations, but with the peculiarity that it can carry over other traits in the process (Burgess et al., 2007; Sheth & Angert, 2016). Because phenotypes are an integration of different trait values that are closely interrelated, they cannot be interpreted independently (Sobral, 2021). In *L. angustifolius*, the advance of flowering onset also involved a change in some of the studied plant traits. We observed that, depending on the populations of origin, the early flowering lines (EFL and OUT) were associated with lower shoot growth, lower biomass, lower height, or lower shoot growth than the control flowering line. These results are in agreement with the hypothesis that we posed related to the resource allocation trade‐offs (Reich, 2014). In other plant species, it has also been found that plants belonging to the early flowering selection lines not only flower earlier but are also smaller and have fewer branches (Burgess et al., 2007; Munguía‐Rosas et al., 2011). Concerning leaf‐structure‐related traits, the EFL had higher LDMC and lower SLA than the CFL, whereas the OUT line had a higher SLA than the CFL. In the first case, the pattern is similar to that found in southern populations with respect to northern populations (Matesanz et al., 2020) and may be related to water‐use efficiency (Rao & Wright, 1994; Wright et al., 1994), indicating that individuals that flower earlier and have lower SLA and higher LDMC could acquire an adaptive advantage in drought environments. On the contrary, the positive association between early flowering and higher SLA of the latter was also found in Sheth and Angert (2016) with *M. cardinalis* and is consistent with a rapid‐growth life‐history strategy (Donovan et al., 2011; Reich, 2014). A possible explanation of some of the differences found between the EFL and the OUT line in some associated traits may rely on the polygenic determination of these traits, the presence of epistatic interactions among genes, and the overdominant expression of some loci associated with heterozygous genotypes (Blümel et al., 2015; Bolger, 2021; He et al., 2019; Holland, 2007). The relevance of these trade‐offs between flowering time and other traits relies in that they may constrain adaptation when genetic correlations are antagonistic to the direction of selection (Etterson & Shaw, 2001; Walsh & Blows, 2009).

The correlations between traits observed in the control line also reveal different adaptive strategies between northern and southern populations, which are reflected in different phenotypic constraints between traits. In northern populations, earlier onset of flowering is correlated with higher biomass, while, in southern populations, earlier flowering is associated with a reduction in plant height and SLA. It is not uncommon for these genetic correlations to vary according to different environmental conditions (Sgrò & Hoffmann, 2004; Sheth & Angert, 2016), because some phenotypic values or combination of traits could have an adaptive advantage in some conditions but not in others (Sobral, 2021). The differences in correlations found between northern and southern populations suggest that northern populations, which have lower water stress, can devote a greater amount of resources to growth, while southern populations must make more efficient use of resources.

### 4.4 Concluding remarks

Artificial selection can allow quantifying genetic variation in traits that are relevant for the adaptation of species to climate change and is useful to determine the adaptive potential of populations. In this study, we experimented with the advancement of flowering time in *L. angustifolius* and found that there are several important aspects to consider. On the one hand, flowering onset of *L. angustifolius* was significantly advanced but only in northern populations. This suggests that northern populations would have a higher capacity for adaptation and that their survival could be higher under the context of climate change, while southern populations would have a higher risk of extinction and would be forced to migrate northward or to higher elevations to track optimal environmental conditions. On the other hand, the advance in flowering onset was found to be associated with changes in other traits, implying that adaptation can be somewhat constrained. These changes were variable depending on the latitude of the populations, implying the existence of different evolutionary constraints to flowering time advancement between northern and southern populations. We did not observe an increase in reproductive success of the early flowering selection lines, probably because these experiments were carried out under controlled conditions without water limitations. We would expect results in natural conditions to provide a fitness advantage to early flowering phenotypes, especially in hot dry years.

In the current times in which climate change is an incipient threat to all organisms, these kinds of studies are essential to assess the risks faced by plant populations and help to better manage and conserve them. Given the exceptional swiftness of climate change, it is unclear whether adaptive evolution can occur fast enough to ensure the survival of all populations. Thus, the insight derived from these studies also sets the stage for the consideration of novel conservation strategies related to assisted evolution. Assisted evolution refers to all strategies in which there is a human intervention in any of the evolutionary forces to help organisms to adapt to environmental conditions (Humanes et al., 2021; Jones & Monaco, 2009). In this sense, artificial selection could increase the prevalence of adaptive alleles of the species that are found in low frequencies in a threatened population, by the reinforcement with individuals containing advantageous alleles (van Oppen et al., 2015). Although these techniques are being tested on some endangered species, such as corals (van Oppen et al., 2015), their application is still incipient, and more in‐depth experimental studies are needed to assess their viability and potential setbacks.

## CONFLICT OF INTEREST

The authors declare that there is no conflict of interest.

## Supporting information


Appendix S1

Appendix S2

## Data Availability

Data associated with this study are made available in the figshare data repository: https://doi.org/10.6084/m9.figshare.21065602 (Sacristán‐Bajo et al., 2022).
