# Supplementary material for: Population origin determines the adaptive potential for the advancement of flowering onset in Lupinus angustifolius L. (Fabaceae)
Source: Evol Appl. 2022 Nov 29;16(1):62–73. doi: 10.1111/eva.13510 (PMC9850010; doi:10.1111/eva.13510)
Supplement: Supplementary file 1 — Appendix S1 [file EVA-16-62-s001.docx]

*Supplementary material 1:*

Detailed protocol of the manual crosses carried out with *Lupinus angustifolius* L.

- Phase 1 – Emasculation: in the inflorescence, we chose the flowers that had not yet opened and had not been self-pollinated. With the help of tweezers disinfected with alcohol, we separated the petals and removed the anthers, taking care not to damage the rest of the structures. We marked the emasculated flowers with a permanent marker pen, and we covered the inflorescence with an organza bag to avoid pollination by pollinators.
- Phase 2 – Pollination: the day after emasculation the flowers were manually pollinated with mature pollen of opened flowers. We removed the pollen-donors flowers from the inflorescence and holding the flower by the calyx, we pressed it, forcing the pollen come out. Then, we deposited the pollen on the stigma of the previously emasculated recipient flower. Finally, we covered the inflorescence again with the organza bag.
