# Supplementary material for: Population origin determines the adaptive potential for the advancement of flowering onset in Lupinus angustifolius L. (Fabaceae)
Source: Evol Appl. 2022 Nov 29;16(1):62–73. doi: 10.1111/eva.13510 (PMC9850010; doi:10.1111/eva.13510)
Supplement: Supplementary file 2 — Appendix S2 [file EVA-16-62-s002.docx]

*Supplementary material 2:*

Table S1. Sample sizes (number of individuals) per population at the flowering time for each selection line and year in which they flowered in the common garden experiments of *Lupinus angustifolius*. CFL: control flowering line; EFL: early flowering line (self-pollinated), OUT: outbred line (cross of different EFL genotypes); OUTS: F2 generation of outbred line resulting from the self-pollination of OUT genotypes.

|  | 2016-2017 | 2017-2018 | | 2018-2019 | | | 2019-2020 | | |
| --- | --- | --- | --- | --- | --- | --- | --- | --- | --- |
| Population | Original line | CFL | EFL | CFL | EFL | OUT | CFL | EFL | OUTS |
| FRO | 193 | 87 | 84 | 63 | 83 | 37 | 46 | 46 | 50 |
| PIC | 193 | 82 | 88 | 63 | 86 | 40 | 44 | 44 | 55 |
| GAR | 194 | 87 | 93 | 84 | 89 | 47 | 46 | 52 | 60 |
| RIV | 191 | 83 | 91 | 84 | 85 | 42 | 44 | 50 | 60 |

Table S2. Effect of selection line, latitude, and year on flowering onset, number of seeds, seed weight, height, biomass, shoot growth, SLA, and LDMC of *Lupinus angustifolius* plants grown in a common garden experiment. Estimates and significance level for fixed effects are shown. Population and Genotype were included as random factors. CFL: control flowering line; EFL: early flowering line (self-pollinated), OUT: outbred line (cross of different EFL genotypes); OUTS: F2 generation of outbred line resulting from the self-pollination of OUT genotypes. Missing factors (CFL = Control line, Latitude North, Year 2018, and interaction CFL: North) are included in the intercept. SLA and LDMC were analyzed separately for each year because they had significant year x line interactions.

| Fixed effects | Estimate | Standard error | t value | p value | Pseudo-R^2^ (fixed effects) | Pseudo- R^2^ (total) |
| --- | --- | --- | --- | --- | --- | --- |
| Flowering onset | - | - | - | - | 0.589 | 0.602 |
| Intercept | 5.017 | 0.012 | 423.927 | <0.001 | - | - |
| EFL | -0.036 | 0.006 | -5.695 | <0.001 | - | - |
| OUT | -0.043 | 0.012 | -3.594 | <0.001 | - | - |
| OUTS | -0.048 | 0.011 | -4.259 | <0.001 | - | - |
| South | 0.118 | 0.016 | -7.198 | <0.001 | - | - |
| 2019 | -0.179 | 0.005 | -36.544 | <0.001 | - | - |
| 2020 | -0.218 | 0.006 | -36.995 | <0.001 | - | - |
| EFL:South | 0.021 | 0.009 | 2.302 | 0.021 | - | - |
| OUT:South | 0.054 | 0.016 | 3.393 | 0.001 | - | - |
| OUTS:South | 0.042 | 0.014 | 2.901 | 0.004 | - | - |
| Number of seeds | - | - | - | - | 0.187 | 0.221 |
| Intercept | 1172.085 | 82.832 | 14.150 | 0.001 | - | - |
| EFL | -51.097 | 48.681 | -1.050 | 0.296 | - | - |
| OUT | -79.296 | 74.353 | -1.066 | 0.287 | - | - |
| South | -591.653 | 113.366 | -5.219 | 0.020 | - | - |
| 2019 | 9.486 | 33.785 | 0.281 | 0.779 | - | - |
| EFL:South | 130.324 | 67.573 | 1.929 | 0.055 | - | - |
| OUT:South | 160.416 | 99.005 | 1.620 | 0.106 | - | - |
| Seed weight | - | - | - | - | 0.497 | 0.627 |
| Intercept | 101.711 | 4.257 | 243.894 | <0.001 | - | - |
| EFL | 5.031 | 2.349 | 2.142 | 0.033 | - | - |
| OUT | 2.363 | 4.059 | 0.582 | 0.561 | - | - |
| South | 46.500 | 5.917 | 7.859 | 0.008 | - | - |
| 2019 | -16.678 | 1.261 | -13.226 | <0.001 | - | - |
| EFL:South | -3.899 | 3.335 | -1.169 | 0.244 | - | - |
| OUT:South | -5.143 | 5.504 | -0.934 | 0.351 | - | - |
| Height | - | - | - | - | 0.409 | 0.589 |
| Intercept | 72.547 | 3.236 | 22.418 | 0.001 | - | - |
| EFL | -1.217 | 0.989 | -1.232 | 0.220 | - | - |
| OUT | -3.038 | 1.587 | -1.941 | 0.057 | - | - |
| South | -16.943 | 4.552 | -3.722 | 0.059 | - | - |
| 2019 | 3.469 | 0.577 | 6.014 | <0.001 | - | - |
| EFL:South | -0.456 | 1.421 | -0.321 | 0.749 | - | - |
| OUT:South | -0.962 | 2.151 | -0.448 | 0.655 | - | - |
| Biomass | - | - | - | - | 0.189 | 0.298 |
| Intercept | 19.239 | 0.737 | 26.114 | <0.001 | - | - |
| EFL | -0.477 | 0.571 | -0.836 | 0.405 | - | - |
| OUT | -3.249 | 0.918 | -3.537 | <0.001 | - | - |
| South | -3.840 | 1.008 | -3.811 | 0.029 | - | - |
| 2019 | -3.496 | 0.340 | -10.296 | <0.001 | - | - |
| EFL:South | 0.522 | 0.803 | 0.651 | 0.517 | - | - |
| OUT:South | 2.238 | 1.222 | 1.832 | 0.068 | - | - |
| Shoot growth | - | - | - | - | 0.387 | 0.683 |
| Intercept | 26.387 | 2.327 | 11.341 | 0.005 | - | - |
| EFL | -4.140 | 0.930 | -4.454 | <0.001 | - | - |
| OUT | -3.191 | 1.466 | -2.177 | 0.031 | - | - |
| South | -13.702 | 3.291 | -4.164 | 0.042 | - | - |
| EFL:South | 1.435 | 1.355 | 1.059 | 0.292 | - | - |
| OUT:South | 4.243 | 2.025 | 2.095 | 0.037 | - | - |
| SLA 2018 | - | - | - | - | 0.007 | 0.194 |
| Intercept | 198.673 | 9.550 | 20.804 | 0.001 | - | - |
| EFL | 0.276 | 4.996 | 0.055 | 0.956 | - | - |
| South | 7.979 | 13.467 | 0.593 | 0.606 | - | - |
| EFL:South | -3.702 | 7.025 | -0.527 | 0.600 | - | - |
| SLA 2019 | - | - | - | - | 0.189 | 0.311 |
| Intercept | 213.305 | 7.952 | 26.823 | <0.001 | - | - |
| EFL | -4.740 | 4.342 | -1.092 | 0.277 | - | - |
| OUT | -3.453 | 5.347 | -0.646 | 0.519 | - | - |
| South | -24.390 | 11.226 | -2.172 | 0.137 | - | - |
| EFL:South | -16.838 | 6.124 | -2.749 | 0.006 | - | - |
| OUT:South | 23.170 | 7.372 | 3.143 | 0.002 | - | - |
| LDMC 2018 | - | - | - | - | 0.249 | 0.320 |
| Intercept | 0.129 | 0.001 | 114.979 | <0.001 | - | - |
| EFL | -0.002 | 0.001 | -1.264 | 0.210 | - | - |
| South | -0.013 | 0.002 | -8.311 | <0.001 | - | - |
| EFL:South | 0.000 | 0.002 | 0.142 | 0.888 | - | - |
| LDMC 2019 | - | - | - | - | 0.054 | 0.105 |
| Intercept | 0.130 | 0.002 | 63.704 | <0.001 | - | - |
| EFL | -0.000 | 0.001 | -0.214 | 0.831 | - | - |
| OUT | -0.004 | 0.002 | -2.158 | 0.032 | - | - |
| South | -0.006 | 0.003 | -2.167 | 0.124 | - | - |
| EFL:South | 0.008 | 0.002 | 3.950 | <0.001 | - | - |
| OUT:South | 0.006 | 0.002 | 2.404 | 0.017 | - | - |

Table S3. Chi-square statistic, degrees of freedom and P-values of the Type II Wald chi-square tests of GLMM and LMM analyses to study the effect of selection line, latitude, and year on flowering onset, number of seeds, seed weight, height, biomass, shoot growth, SLA, and LDMC of *Lupinus angustifolius* plants grown in a common garden experiment. Estimates and significance level for fixed effects are shown. Population and Genotype were included as random factors. CFL: control flowering line; EFL: early flowering line (self-pollinated), OUT: outbred line (cross of different EFL genotypes); OUTS: F2 generation of outbred line resulting from the self-pollination of OUT genotypes. Missing factors (CFL = Control line, Latitude North, Year 2018, and interaction CFL: North) are included in the intercept. SLA and LDMC were analyzed separately for each year because they had significant year x line interactions.

| Fixed effects | *X*^2^ | Df | Pr (>\|*X*^2^\|) |
| --- | --- | --- | --- |
| Flowering onset | - | - | - |
| Line | 35.807 | 3 | <0.001 |
| Year | 1987.443 | 2 | <0.001 |
| Latitude | 41.841 | 1 | <0.001 |
| Line:latitude | 17.298 | 3 | <0.001 |
| Number of seeds | - | - | - |
| Line | 0.254 | 2 | 0.881 |
| Year | 0.079 | 1 | 0.779 |
| Latitude | 22.800 | 1 | <0.001 |
| Line:latitude | 4.679 | 2 | 0.096 |
| Seed weight | - | - | - |
| Line | 3.983 | 2 | 0.137 |
| Year | 174.921 | 1 | <0.001 |
| Latitude | 61.890 | 1 | <0.001 |
| Line:latitude | 1.721 | 2 | 0.423 |
| Height | - | - | - |
| Line | 11.311 | 2 | 0.003 |
| Year | 36.166 | 1 | <0.001 |
| Latitude | 14.944 | 1 | <0.001 |
| Line:latitude | 0.232 | 2 | 0.890 |
| Biomass | - | - | - |
| Line | 10.641 | 2 | 0.005 |
| Year | 106.004 | 1 | <0.001 |
| Latitude | 13.090 | 1 | <0.001 |
| Line:latitude | 3.358 | 2 | 0.187 |
| Shoot growth | - | - | - |
| Line | 27.139 | 2 | <0.001 |
| Latitude | 14.572 | 1 | <0.001 |
| Line:latitude | 4.510 | 2 | 0.105 |
| SLA 2018 | - | - | - |
| Line | 0.207 | 1 | 0.649 |
| Latitude | 0.220 | 1 | 0.639 |
| Line:latitude | 0.278 | 1 | 0.598 |
| SLA 2019 | - | - | - |
| Line | 41.084 | 2 | <0.001 |
| Latitude | 5.705 | 1 | 0.017 |
| Line:latitude | 30.247 | 2 | <0.001 |
| LDMC 2018 | - | - | - |
| Line | 2.705 | 1 | 0.100 |
| Latitude | 126.822 | 1 | <0.001 |
| Line:latitude | 0.020 | 1 | 0.887 |
| LDMC 2019 | - | - | - |
| Line | 41.083 | 2 | <0.001 |
| Latitude | 5.705 | 1 | 0.016 |
| Line:latitude | 30.257 | 2 | <0.001 |

Table S4. Posterior mean values, standard errors, and 95 % confidence intervals for the different traits and lines of *Lupinus angustifolius* plants grown in a common garden experiment. CFL: control flowering line; EFL: early flowering line (self-pollinated), OUT: outbred line (cross of different EFL genotypes); OUTS: F2 generation of outbred line resulting from the self-pollination of OUT genotypes.

|  | North | | | | South | | | |
| --- | --- | --- | --- | --- | --- | --- | --- | --- |
|  | Mean | Std. error | 2∙5% | 97∙% | Mean | Std. error | 2∙5% | 97∙% |
| Flowering onset | - | - | - | - | - | - | - | - |
| CFL | 132 | 1.54 | 129 | 135 | 117 | 1.37 | 115 | 120 |
| EFL | 128 | 1.48 | 125 | 130 | 116 | 1.34 | 113 | 118 |
| OUT | 127 | 1.94 | 123 | 131 | 119 | 1.78 | 115 | 122 |
| OUTS | 126 | 1.84 | 123 | 130 | 117 | 1.69 | 114 | 120 |
| Number of seeds | - | - | - | - | - | - | - | - |
| CFL | 1177 | 80.80 | 900 | 1456 | 585 | 79.70 | 300 | 871 |
| EFL | 1126 | 79.1 | 836 | 1415 | 664 | 79.20 | 375 | 953 |
| OUT | 1098 | 97.1 | 855 | 1340 | 666 | 93.90 | 422 | 911 |
| Seed weight | - | - | - | - | - | - | - | - |
| CFL | 93.40 | 4.21 | 78.60 | 108 | 139.90 | 4.16 | 124.80 | 155 |
| EFL | 98.40 | 4.17 | 83.40 | 113 | 141.00 | 4.15 | 125.90 | 156 |
| OUT | 95.70 | 5.11 | 83.00 | 108 | 137.10 | 4.96 | 124.30 | 150 |
| Height | - | - | - | - | - | - | - | - |
| CFL | 74.30 | 3.22 | 61.30 | 87.30 | 57.30 | 3.22 | 44.30 | 70.40 |
| EFL | 73.10 | 3.21 | 60.00 | 86.10 | 55.70 | 3.21 | 42.60 | 68.80 |
| OUT | 71.20 | 3.41 | 59.60 | 82.80 | 53.30 | 3.37 | 41.50 | 65.20 |
| Biomass | - | - | - | - | - | - | - | - |
| CFL | 17.50 | 0.72 | 15.30 | 19.70 | 13.70 | 0.71 | 11.04 | 15.90 |
| EFL | 17.00 | 0.71 | 14.80 | 19.20 | 13.70 | 0.70 | 11.40 | 15.90 |
| OUT | 14.20 | 0.98 | 12.10 | 16.40 | 12.60 | 0.92 | 10.60 | 14.70 |
| Shoot growth | - | - | - | - | - | - | - | - |
| CFL | 26.39 | 2.33 | 17.47 | 35.30 | 12.69 | 2.33 | 3.77 | 21.60 |
| EFL | 22.25 | 2.32 | 13.28 | 31.20 | 9.98 | 2.31 | 0.95 | 19.00 |
| OUT | 23.20 | 2.50 | 15.29 | 31.10 | 13.74 | 2.46 | 5.66 | 21.80 |
| SLA 2018 | - | - | - | - | - | - | - | - |
| CFL | 199 | 9.50 | 163 | 235 | 207 | 9.50 | 170 | 243 |
| EFL | 199 | 9.50 | 163 | 235 | 203 | 9.50 | 167 | 240 |
| SLA 2019 | - | - | - | - | - | - | - | - |
| CFL | 213 | 7.95 | 185 | 242 | 189 | 7.93 | 160 | 218 |
| EFL | 209 | 7.92 | 180 | 237 | 167 | 7.90 | 138 | 196 |
| OUT | 210 | 8.43 | 184 | 236 | 209 | 8.28 | 182 | 235 |
| LDMC 2018 | - | - | - | - | - | - | - | - |
| CFL | 0.13 | 0.00 | 0.13 | 0.13 | 0.12 | 0.00 | 0.11 | 0.12 |
| EFL | 0.13 | 0.00 | 0.12 | 0.13 | 0.11 | 0.00 | 0.11 | 0.12 |
| LDMC 2019 | - | - | - | - | - | - | - | - |
| CFL | 0.13 | 0.00 | 0.12 | 0.14 | 0.12 | 0.00 | 0.12 | 0.13 |
| EFL | 0.13 | 0.00 | 0.12 | 0.14 | 0.13 | 0.00 | 0.13 | 0.14 |
| OUT | 0.13 | 0.00 | 0.12 | 0.13 | 0.13 | 0.00 | 0.12 | 0.13 |

Table S5: Observed mean ±SD values for the different traits measured and the different lines tested. SLA: specific leaf area, LDMC: leaf dry matter content. CFL: control flowering line; EFL: early flowering line (self-pollinated), OUT: outbred line (cross of different EFL genotypes); OUTS: F2 generation of outbred line resulting from the self-pollination of OUT genotypes.

|  | Flowering onset (days) | | | | Number of seeds | | | Seed weight (mg) | | | Total height (cm) | | | Shoot growth (cm) | | | SLA (cm^2^/g) | | | LDMC (mg/g) | | | Biomass (g) | | |
| --- | --- | --- | --- | --- | --- | --- | --- | --- | --- | --- | --- | --- | --- | --- | --- | --- | --- | --- | --- | --- | --- | --- | --- | --- | --- |
|  | CFL | EFL | OUT | OUTS | CFL | EFL | OUT | CFL | EFL | OUT | CFL | EFL | OUT | CFL | EFL | OUT | CFL | EFL | OUT | CFL | EFL | OUT | CFL | EFL | OUT |
| North | 136.06  ±15.33 | 130.26  ±15.28 | 120.92  ±7.54 | 115.66  ±9.02 | 1180.99  ±559.19 | 1123.60  ±693.73 | 1101.45  ±572.63 | 92.37  ±23.64 | 97.71  ±24.70 | 87.78  ±17.74 | 74.96  ±10.33 | 72.90  ±11.35 | 72.79  ±11.26 | 27.29  ±9.71 | 21.14  ±8.05 | 22.48  8.47 | 206.14  ±35.44 | 203.96  ±36.17 | 209.66  ±44.44 | 0.13  ±0.01 | 0.13  ±0.01 | 0.13  ±0.01 | 17.63  ±7.30 | 16.96  ±6.88 | 12.55  ±4.98 |
| FRO | 136.12  ±14.73 | 130.85  ±14.70 | 124.32  ±5.73 | 118.90  ±8.24 | 1297.12  ±626.99 | 1208.61  ±720.90 | 1179.69  ±576.11 | 96.15  ±22.48 | 100.45  ±24.86 | 91.44  ±19.11 | 74.29  ±9.74 | 74.89  ±10.56 | 74.77  ±11.78 | 26.39  ±8.64 | 23.05  ±8.06 | 27.14  ±6.84 | 205.89  ±39.19 | 202.58  ±32.47 | 207.28  ±39.22 | 0.13  ±0.01 | 0.13  ±0.01 | 0.13  ±0.01 | 17.93  ±7.55 | 18.52  ±6.88 | 13.76  ±5.14 |
| PIC | 135.99  ±15.96 | 129.67  ±15.83 | 117.69  ±7.70 | 112.72  ±8.75 | 1056.97  ±446.93 | 1044.09  ±660.03 | 1025.39  ±566.86 | 88.35  ±24.28 | 95.05  ±24.35 | 84.54  ±16.01 | 75.70  ±10.94 | 71.03  ±11.77 | 70.92  ±8.93 | 28.16  ±10.65 | 19.23  ±7.63 | 18.08  ±7.51 | 206.41  ±31.01 | 205.26  ±39.41 | 211.86  ±49.17 | 0.13  ±0.01 | 0.13  ±0.01 | 0.12  ±0.02 | 17.31  ±7.05 | 15.38  ±6.54 | 11.34  ±4.57 |
| South | 120.02  ±13.13 | 117.98  ±12.83 | 113.57  ±6.18 | 107.23  ±7.54 | 589.29  ±434.47 | 665.04  ±429.37 | 670.70  ±331.97 | 139.52  ±24.45 | 140.36  ±26.12 | 128.28  ±22.15 | 57.85  ±9.69 | 55.46  ±9.45 | 55.45  10.00 | 12.53  ±7.18 | 9.64  ±5.09 | 14.03  ±6.48 | 197.25  ±39.47 | 184.18  ±38.05 | 209.35  ±36.26 | 0.12  ±0.01 | 0.12  ±0.01 | 0.13  ±0.01 | 13.73  ±5.19 | 13.36  ±4.23 | 10.91  ±4.07 |
| GAR | 122.52  ±13.29 | 120.62  ±12.64 | 116.30  ±4.43 | 108.37  ±6.82 | 535.67  ±395.07 | 574.34  ±409.68 | 759.19  ±315.38 | 140.59  ±23.64 | 147.13  ±28.92 | 138.88  ±20.73 | 61.38  ±9.81 | 59.56  ±8.92 | 61.99  ±7.63 | 15.50  ±7.72 | 12.41  ±4.61 | 16.95  ±5.81 | 210.12  ±44.93 | 195.87  ±39.12 | 216.08  ±28.70 | 0.12  ±0.01 | 0.12  ±0.01 | 0.13  ±0.01 | 12.78  ±5.07 | 13.30  ±4.05 | 12.08  ±4.06 |
| RIV | 117.47  ±12.49 | 115.24  ±12.48 | 110.52  ±6.46 | 106.05  ±8.12 | 636.41  ±462.73 | 754.40  ±431.04 | 582.20  ±328.01 | 138.37  ±18.34 | 133.60  ±20.98 | 117.40  ±18.10 | 54.18  ±8.10 | 51.58  ±8.25 | 47.93  ±6.51 | 9.41  ±4.97 | 7.29  ±4.23 | 10.66  ±5.55 | 183.97  ±27.29 | 172.75  ±33.33 | 201.82  ±36.62 | 0.12  ±0.01 | 0.12  ±0.01 | 0.13  ±0.01 | 14.70  ±5.14 | 13.41  ±4.40 | 9.69  ±3.76 |


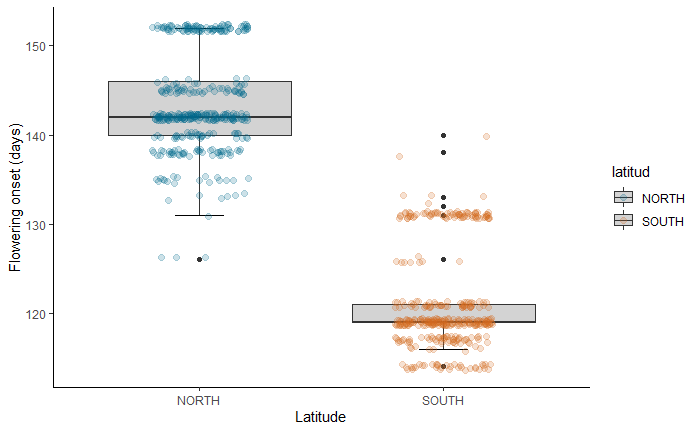


Figure S1. Flowering onset of plants of *Lupinus angustifolius* L. from northern (Zarapicos and Zafrón) and sourthern (La Garranchosa and Rivera de la Lanchita) populations in 2017.


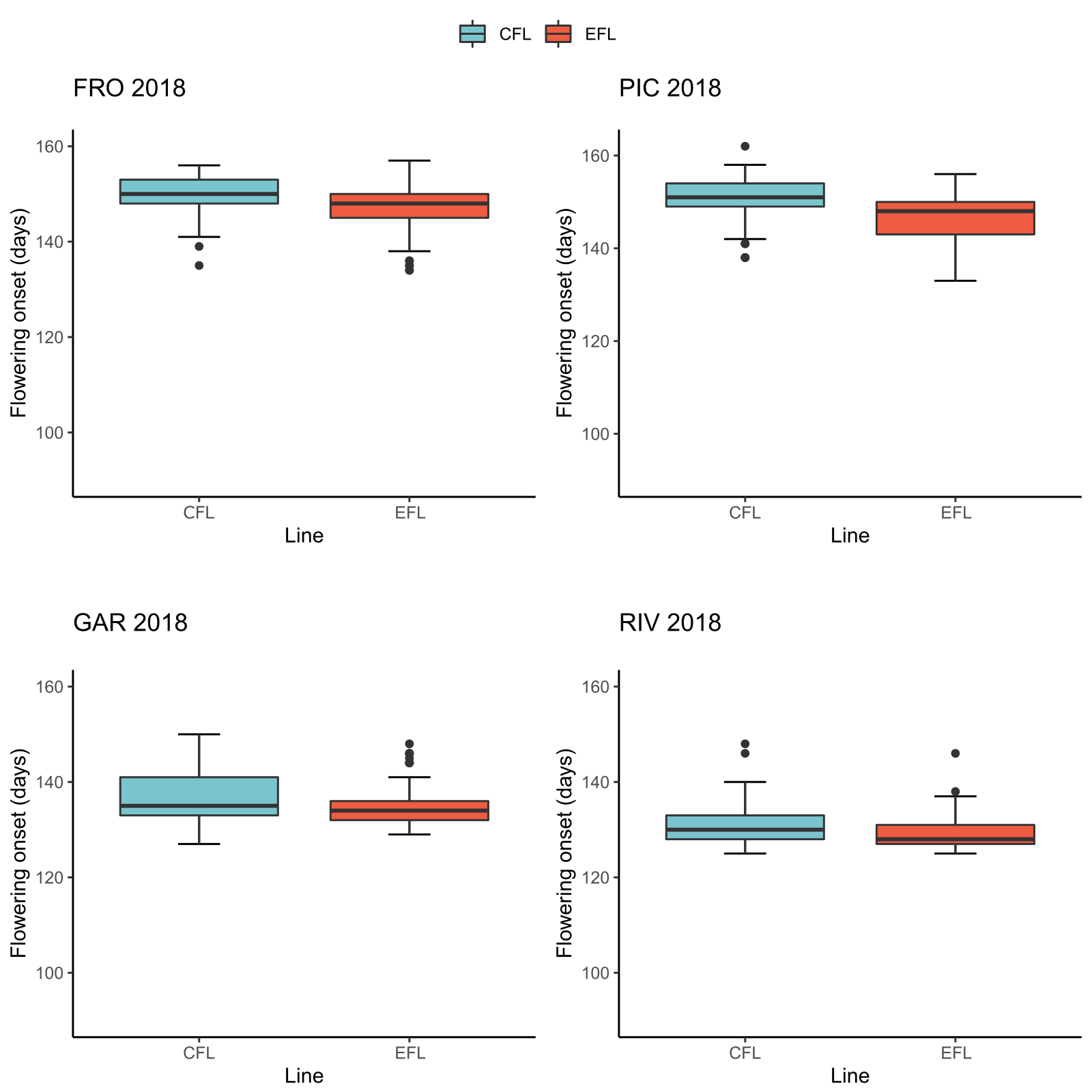


Figure S2. Observed values of flowering onset of *Lupinus angustifolius* for the different populations in the year 2018.


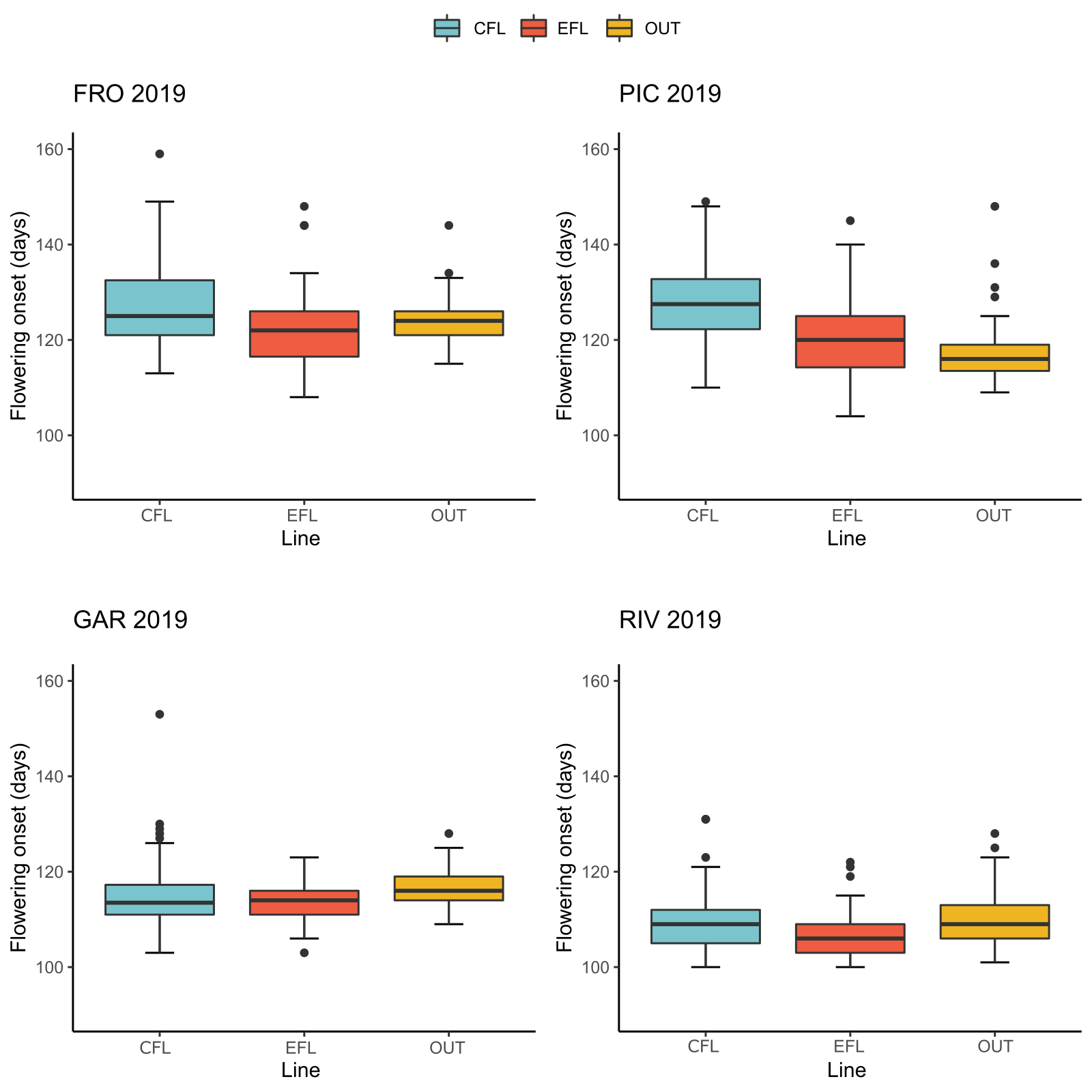


Figure S3. Observed values of flowering onset of *Lupinus angustifolius* for the different populations in the year 2019.


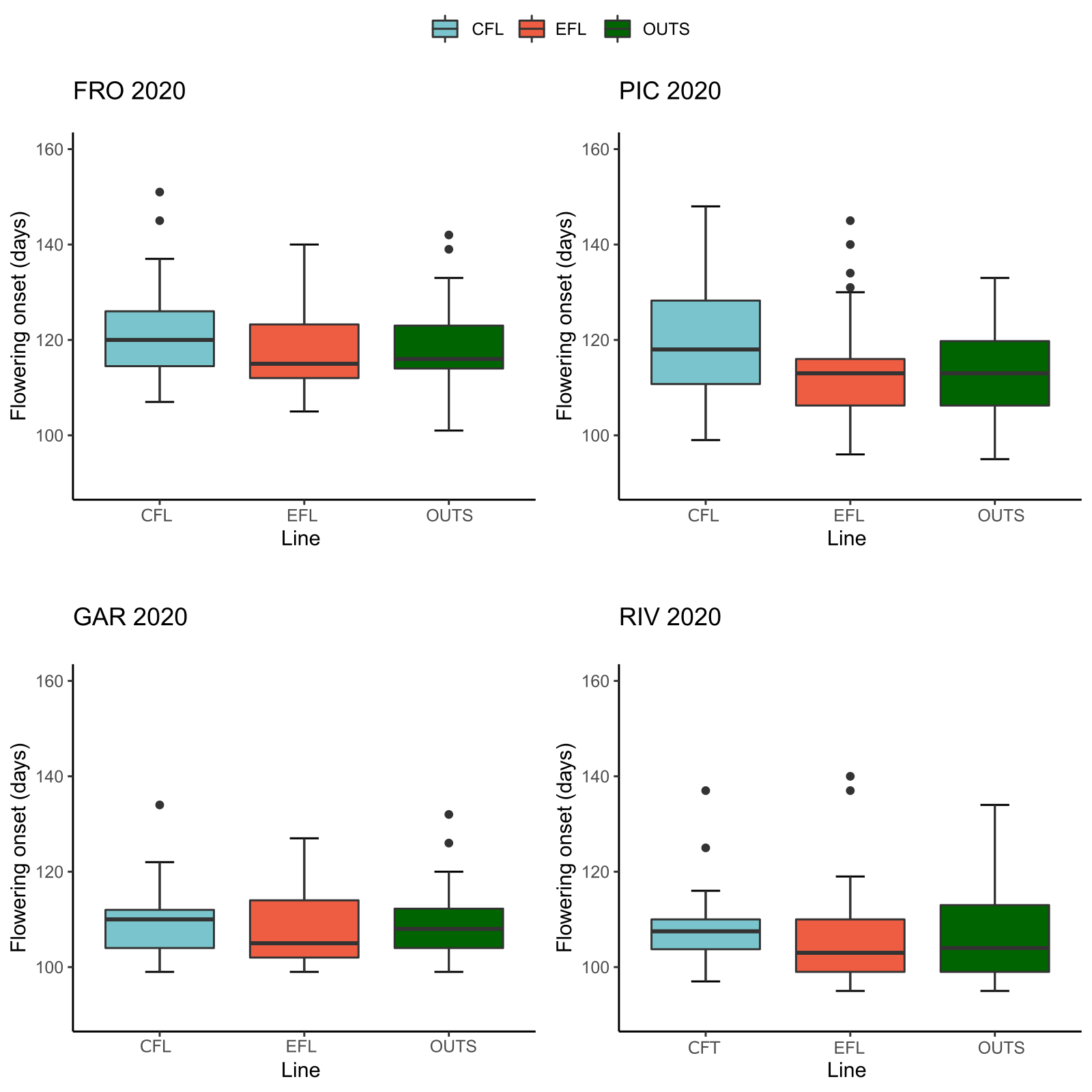


Figure S4. Observed values of flowering onset of *Lupinus angustifolius* for the different populations in the year 2020.


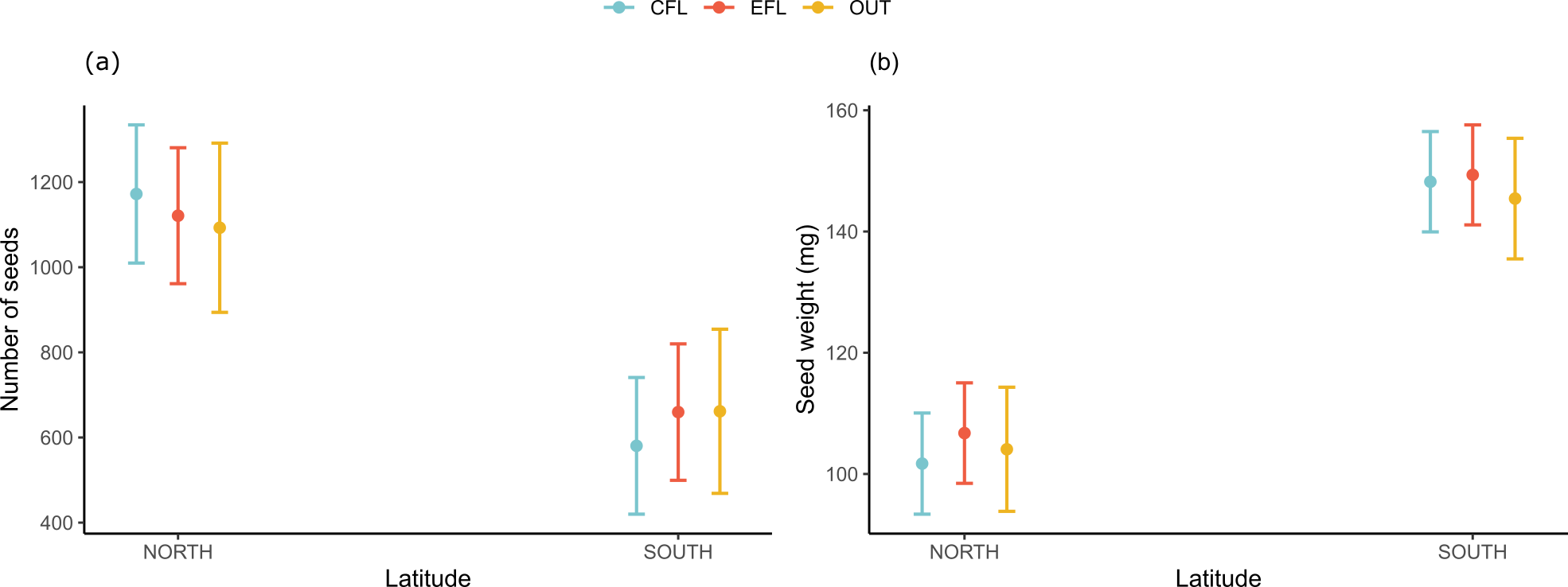


Figure S5. Effect of artificial selection lines (EFL and OUT) on fitness components in northern and southern populations of *Lupinus angustifolius* L.: (a) number of seeds per plant and (b) mean seed weight. Years are analyzed together. CFL: control flowering line; EFL: early flowering line (self-pollinated), OUT: outbred line (cross of different EFL genotypes); OUTS: F2 generation of outbred line resulting from the self-pollination of OUT genotypes. Dots and bars represent the predicted mean from the LMM model with a Gaussian distribution and the 95 % confidence intervals. Differences between the artificial selection lines and the control line were non-significant.
